# Supplementary material for: Perfluorocarbon Nanodroplets as Potential Nanocarriers for Brain Delivery Assisted by Focused Ultrasound-Mediated Blood–Brain Barrier Disruption
Source: Pharmaceutics. 2022 Jul 19;14(7):1498. doi: 10.3390/pharmaceutics14071498 (PMC9323719; doi:10.3390/pharmaceutics14071498)
Supplement: Supplementary file 1 [file pharmaceutics-14-01498-s001.zip › pharmaceutics-1785556-Supplementary.pdf]

pharmaceutics

Supplementary Materials

Perfluorocarbon Nanodroplets as Potential Nanocarriers for Brain Delivery Assisted by Focused Ultrasound-Mediated Blood–Brain Barrier Disruption

Charlotte Bérard [1,†], Stéphane Desgranges [2,†], Noé Dumas [3], Anthony Novell [4], Benoit Larrat [5], Mourad Hamimed [6], Nicolas Taulier [7], Marie-Anne Estève [1,*], Florian Correard [1,‡] and Christiane Contino-Pépin [2,*,‡]

1 Aix Marseille Univ, APHM, CNRS, INP, Inst Neurophysiopathol, Hôpital Timone, Service Pharmacie, Marseille, France; charlotte.berard@ap-hm.fr (C.B.); florian.correard@univ-amu.fr (F.C.)
2 Avignon Université, Unité Propre de Recherche et d'Innovation,Équipe Systèmes Amphiphiles bioactifs et Formulations Eco-compatibles, 84000 Avignon, France; stephane.desgranges@univ-avignon.fr (S.D.)
3 Aix Marseille Univ, CNRS, INP, Inst Neurophysiopathol, Marseille, France; noe.dumas@gmail.com (N.D.)
4 Université Paris-Saclay, CEA, CNRS, Inserm, BioMaps, Service Hospitalier Frédéric Joliot, 91401 Orsay, France; anthony.novell@universite-paris-saclay.fr (A.N.)
5 Université Paris Saclay, CEA, CNRS, NeuroSpin / BAOBAB, 91191 Gif-sur-Yvette, France; benoit.larrat@cea.fr (B.L.)
6 Aix Marseille Univ, CNRS, INSERM, Institut Paoli-Calmettes, CRCM, SMARTc Unit, COMPO Inria – Inserm project team, Marseille, France; mourad.hamimed@univ-amu.fr (M.H.)
7 Sorbonne Université, CNRS, INSERM, Laboratoire d'Imagerie Biomédicale—LIB, 75006 Paris, France; nicolas.taulier@sorbonne-universite.fr (N.T.)
* Correspondence: marie-anne.esteve@univ-amu.fr (M.-A.E.); christine.pepin@univ-avignon.fr (C.C.-P.)
† These authors contributed equally to this work.
‡ These authors contributed equally to this work.

Table S1. Titration of oil and PFOB by HPLC and ^{19}F-NMR before and after emulsion filtration, respectively.

	ATBC Before filtration	ATBC After filtration	C-90 Before filtration	C-90 After filtration
PFOB % (v/v)	3.92±0.25	3.88±0.41	3.88±0.29	3.75±0.16
Oil µl mL^{-1a}	4.53±0.16	3.45±0.09	8.13±0.07	7.38±0.25
% (v/v) of oil in $\Phi_{dispersed}$^{b}	10.04±0.16	8.22±0.45	17.4±0.77	16.2±0.37

^{a}µL mL^{-1} of emulsion; ^{b}dispersed phase ($\Phi_{dispersed} = V_{oil} + V_{PFOB}$).

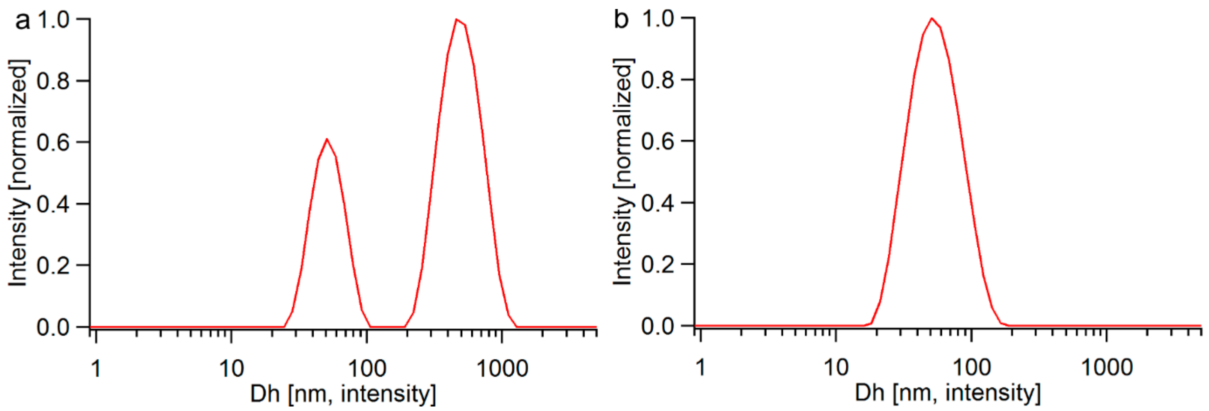

Figure S1. DLS of emulsion with 10% ATBC before (a) and after (b) filtration (Intensity weighted distribution).

Table S2. Freeze-drying assay with a Fv of 4.7% (with 5% ATBC) and with a Fv of 9.1% (no oil).

	**% (w/v)[a] of Trehalose**	**Dh [nm]**		**S [Sf/Si][b]**		**% [v/v] PFOB remaining**	
		No oil	5% ATBC	No oil	5% ATBC	No oil	5% ATBC
Baseline formulation before FD process	0	63±1	52±2				
Baseline formulation after FD process	0	159±5	77±18	2.52	1.50	74.0±5.5%	101.4±1.4%
	5%	77±3	54±4	1.22	1.05	89.1±1.8%	104.4±3.6%
	10%	75±4	55±3	1.20	1.06	82.7±4.8%	99.9±4.7%
	15%	78±5	58±3	1.24	1.13	84.9±4.3%	93.9±8.8%
	20%	82±3	58±4	1.30	1.12	78.4±4.4%	100.3±3.1%
	25%	85±3	60±4	1.34	1.16	78.7±0.3%	91.0±9.9%

[a] % of Trehalose after emulsion rehydration; [b] Si: Droplet Dh after preparation (T_0), Sf: Droplet Dh after rehydration.

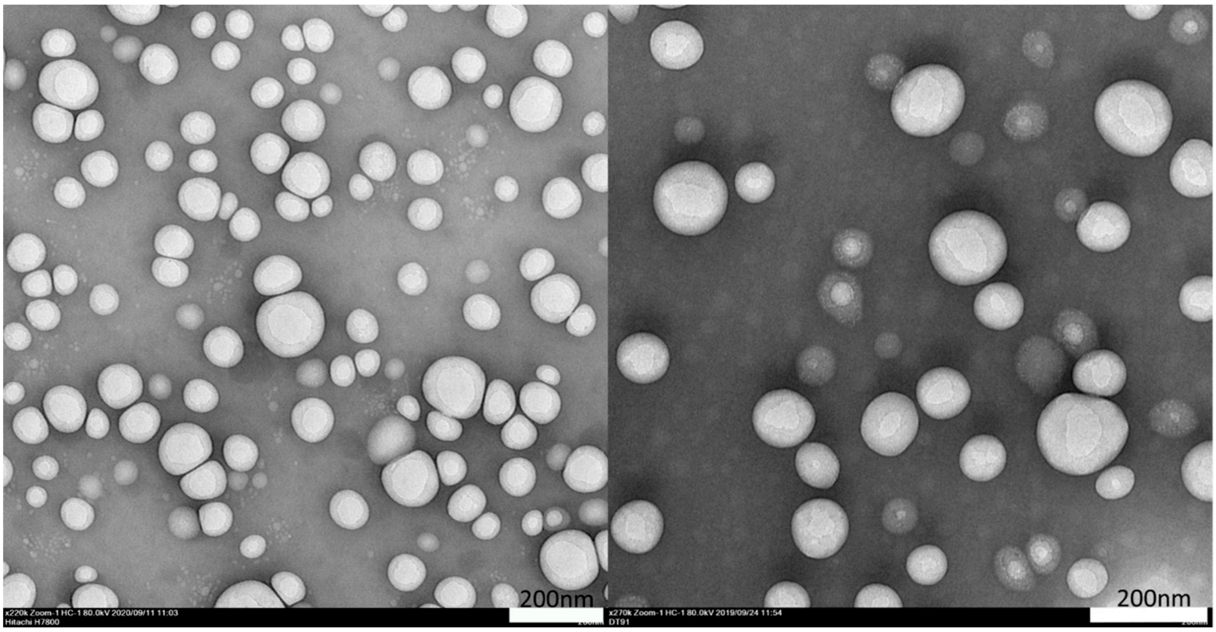

Figure S2. TEM images of emulsion after rehydration: STE emulsion (75±24 nm) (left); DTE emulsion (86±23 nm) (right). Size determined using Image J.

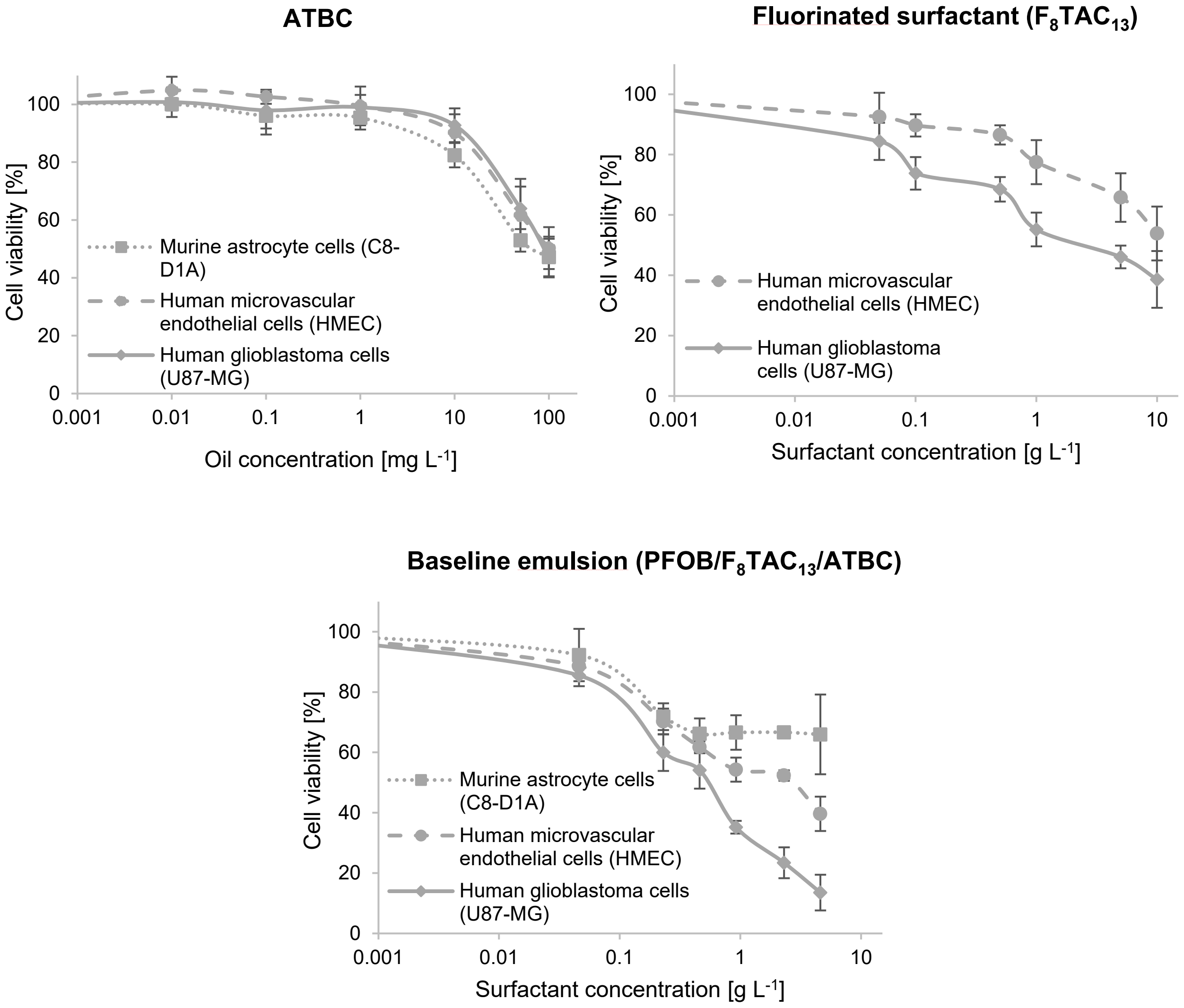

Figure S3. *In vitro* biocompatibility assessment of nanoemulsion and its components by MTT assay on three cell lines incubated for 72 h with ATBC (0.01 - 100 mg L^{-1}) (a), F_8TAC_{13} surfactant (0.05 - 10 g L^{-1}) (b) and baseline emulsion (0.046 - 4.6 g L^{-1} of F_8TAC_{13}) (c). The values are expressed as mean ± SD (n = 3). MTT, 3-(4,5-dimethylthiazol-2-yl)-2,5-diphenyltetrazolium bromide.

Table S3. Summary of PFOB concentrations in blood assessed by ^{19}F-NMR for each sampling time-point (21 records below the LLOQ were excluded: one mouse at the 24 h sampling point and all mice at the 48 h to 14 days sampling points).

**Time [h]**	**n**	**PFOB concentration [µL mL^{-1} blood]**				
		median	Q1	Q3	mean	SD
0.083	5	21.66	18.71	21.79	20.37	2.38
0.25	5	22.50	22.38	24.75	23.08	1.89
0.5	5	24.23	22.70	25.38	24.00	1.91
1	5	22.51	21.70	23.34	20.49	5.80
2	5	25.90	24.20	27.27	26.10	2.80
4	5	18.70	18.37	19.89	19.16	1.40
8	5	20.42	16.29	21.36	19.15	2.67
16	3[a]	3.63	3.46	3.81	3.64	0.35
24	4	0.58	0.21	1.46	1.09	1.32

[a] 2 mice died.

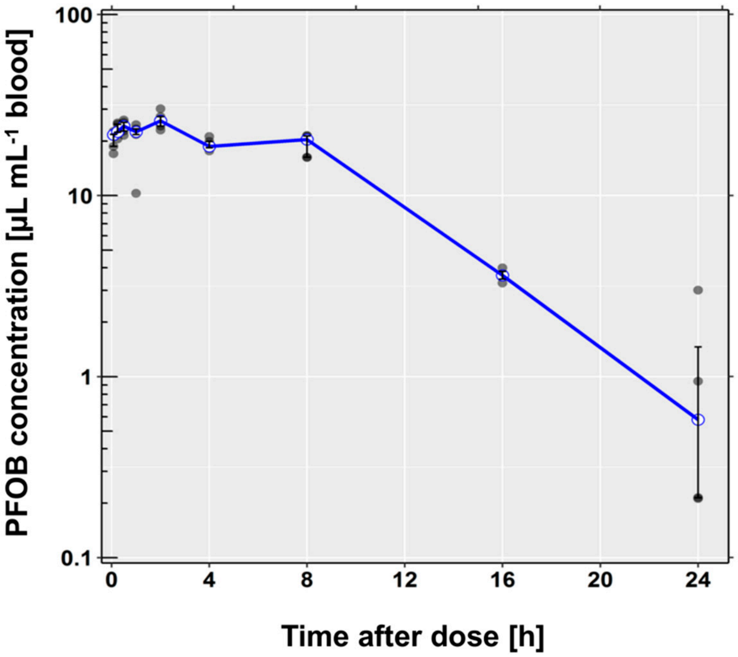

Figure S4. Median (interquartile range) concentration-time profile of PFOB in blood on semi-logarithmic scale, overlaid with observed data points, after blood sampling 5, 15, 30, 60 min, 2, 4, 8, 16 and 24 h following a retro-orbital i.v. administration of a single dose of 300 µL of the concentrated emulsion.
